# Development of laser induced breakdown spectroscopy technique to study irrigation water quality impact on nutrients and toxic elements distribution in cultivated soil

**DOI:** 10.1016/j.sjbs.2021.07.064

**Published:** 2021-07-29

**Authors:** I. Rehan, M.A. Gondal, R.K. Aldakheel, K. Rehan, S. Sultana, M.A. Almessiere, Z. Ali

**Affiliations:** a Department of Physics, Islamia College University, Peshawar 25120, Pakistan; b Physics Department, IRC-Hydrogen and Energy Storage, K.A.CARE Energy Research and Innovation Center, King Fahd University of Petroleum and Minerals, P.O. Box 5047, Dhahran 31261, Saudi Arabia; c Department of Physics, College of Science, Imam Abdulrahman Bin Faisal University, Dammam P.O. Box 1982, Saudi Arabia; d Department of Biophysics, Institute for Research and Medical Consultations (IRMC), Imam Abdulrahman Bin Faisal University, Dammam P.O. Box 1982, Saudi Arabia; e CAS Key Laboratory of Microscale Magnetic, University of Science and Technology of China, Hefei, Anhui, 230026, PR China; f Department of Chemistry, Islamia College University, Peshawar 25120, Pakistan; g Nano science and catalysis division, National center for Physics, Islamabad Pakistan

**Keywords:** CF-LIBS, CC-LIBS, ICP-OES, Cultivated soils, LIBS Soil analysis, Water management for land cultivation

## Abstract

This study is focused mainly on impact of irrigation water quality in cultivated soil on distribution of essentials nutrients (Al, Mg, Ca, Fe, S, Si, Na, P, and K) and relatively toxic metals (As, Ba, Cr, Cu, Ti, Sn, Mn, Ni, and Zn) using an elegant Laser induced breakdown spectroscopy (LIBS) technique. A pulsed Nd:YAG laser operating at 1064 nm in conjunction with suitable detector was applied to record soil emission spectra. The abundance of these elements were evaluated via standard calibration curve Laser Induced Breakdown Spectroscopy (CC-LIBS) and calibration free Laser Induced Breakdown Spectroscopy (CF-LIBS) approaches. Quantitative analyses were accomplished under conjecture of local thermodynamic equilibrium (L.T.E) and optically thin plasma. The average electron temperatures were estimated by Boltzmann plot method for cultivated soil samples in 7800 to 9300 K range. The electron number density was ~ 1.11 × 1017 cm − 3 to 1.60 × 1017 cm − 3. Prior to application on soil samples, the experimental setup was optimized at the following parameters: pulsed energy = 60 mJpulse-1, sample to lens distance of 9.0 cm, and the gate delay of 3.5 μs. It is noteworthy that nutritional elements content of cultivated soils were found strongly dependent upon the irrigation water quality. The cultivated soil from industrial area was found rich of toxins while the cultivated land using tube well water contains toxins in least amount. Our LIBS findings were also validated by comparing its results with contents measured using a standard inductively coupled plasma optical emission spectroscopy (ICP-OES) method and both were found in excellent agreement. The present study could be highly beneficial for agricultural applications and for farmers to produce safe food products and higher crops yield.

## 1 Introduction

At present, the humanity is facing numerous challenges associated to ensure safe supplies of foodstuff for the rapid growing population in addition to the potential use of cultivable land for biofuel production (Schmoldt, 2001). The cultivable land contained nutritional as well as some toxic metals. Essential or nutrient elements are chemical constituents which are prerequisites for vegetation to grow and replicate. Metals are basic constituents of the earth's layers; nowadays contamination of soil with toxic metals due to environmental issues are being faced by the humanity globally and this problem is becoming more and more stringent with the upsurge of industrialization (Hussain et al., 2013). The chemical composition of agricultural plants is greatly dependent upon the composition of cultivated soil. In different areas of the world, the local farmers for watering their crops use irrigation water from different sources like tube wells, canal water and rainy water etc. Many countries, comprising Pakistan and India, farmers irrigates their crops with industrial discharge (Rattan et al., 2005, Kaushik et al., 2005) owing to the non-availability of substitute sources of irrigation water (Kumar and Chandra, 2004, Tiwari et al., 2008). Any irrigated agricultural plant essentials can be classified as macro essentials (like potassium (K), calcium (Ca), sulfur (S), magnesium (Mg), and phosphorus (P)), micro-nutrients (iron (Fe), copper (Cu), molybdenum (Mb), nickel (Ni), zinc (Zn), and manganese (Mn)), as well as some beneficial species such as (sodium (Na), cobalt (Co), and silicon (Si)) which depend on the quantity absorbed by the plants from the soil. To estimate the concentration of such essential nutrients in agricultural soil is a laborious exercise for the farmers as it requires to collect the soil samples from cultivated fields and then carry them to a specific advanced soil-research laboratory. The farmers can then amend the fertilizing pattern based on the outcomes/results obtained from analytical laboratory which normally takes a period of one to two weeks (Stafford, 2000). There are various techniques for the quantitative analysis of soil samples, but most of these techniques are expensive, time consuming and hence the nutrients data influencing the crop yield could not be improved and optimized in a real time scenario. One of the most powerful analytical tool for this purpose is Laser-induced breakdown spectroscopy (LIBS) which allows one to acquire the plasma emission of every sample i.e. solid, liquid as well as gaseous state (Rehan et al., 2016, Rai et al., 2003), from which one can get the quantitative and qualitative information of the test sample contents by the spectro-chemical analysis. LIBS is an atomic emission spectroscopic method that has been extensively employed for soil analysis, mostly to evaluate the total carbon contents, heavy metals in soil (Cremers et al., 2001, Ding et al., 2019) and to detect the contaminants like Pb in polluted soil (Rehan et al., 2018). This technique utilizes an energetic pulsed laser to produce a micro-plasma at temperatures adequately high for the vaporization and dissociation of target material within the spark region into neutral and ionic particles. After the formation of spark (usually in the first few hundred nano-seconds), collisions between atomic–ionic particles create spectrally wide continuum which dominates the optical emission. After the decay of plasma temperature and electron density (nearly 0.5–2.0 μs after ignition of spark), discharge from excited elemental ions dominates the emission spectra. After a time span of around 2–5 μs, the spectral emissions from atomic species become dominant. For precise quantifications of elemental contents of target samples, the optimization of various LIBS experimental parameters is very significant. LIBS provide some important dominance: (i) real-time and in situ examination of matter in any state, (ii) negligible or no sample preparation required, and (iii) the option of multi-elemental and open-field calculations. Taking into consideration the above mentioned facts, LIBS appears to be an appropriate method for real-time and in-situ finding of nutrients distribution in cultivated soils.

The aim of present work is to employ an optimized quantitative LIBS approach to carry out the elemental distributions in cultivated soil samples, specifically aiming to evaluate the effect of irrigating water on soil chemical composition for the improvement of crops yield and also the food quality. For this purpose, cultivated soil samples were collected from KP province of Pakistan. The acquired samples were dried, sieved, crushed to powder, after then pelletized. The quantitative analysis was performed via calibration free (CF)-LIBS and standard calibration curves (CC)-LIBS methods. To make sure the optimum experimental conditions for the precise assessment of elemental composition of irrigated soil, the hypothesis of local thermodynamic equilibrium (L.T.E) and the creation of optically thin plasma (O.T.P) was confirmed by determining the major plasma parameters such as plasma temperature (T) as well as number density (N*_e_*). Furthermore, the quantification results obtained using LIBS was confirmed by a standard ICP-OES analytical approach and it is worth mentioning that LIBS and ICP-OES results were in good agreement.

## 2 Details of LIBS experimental setup

**Fig. 1:**
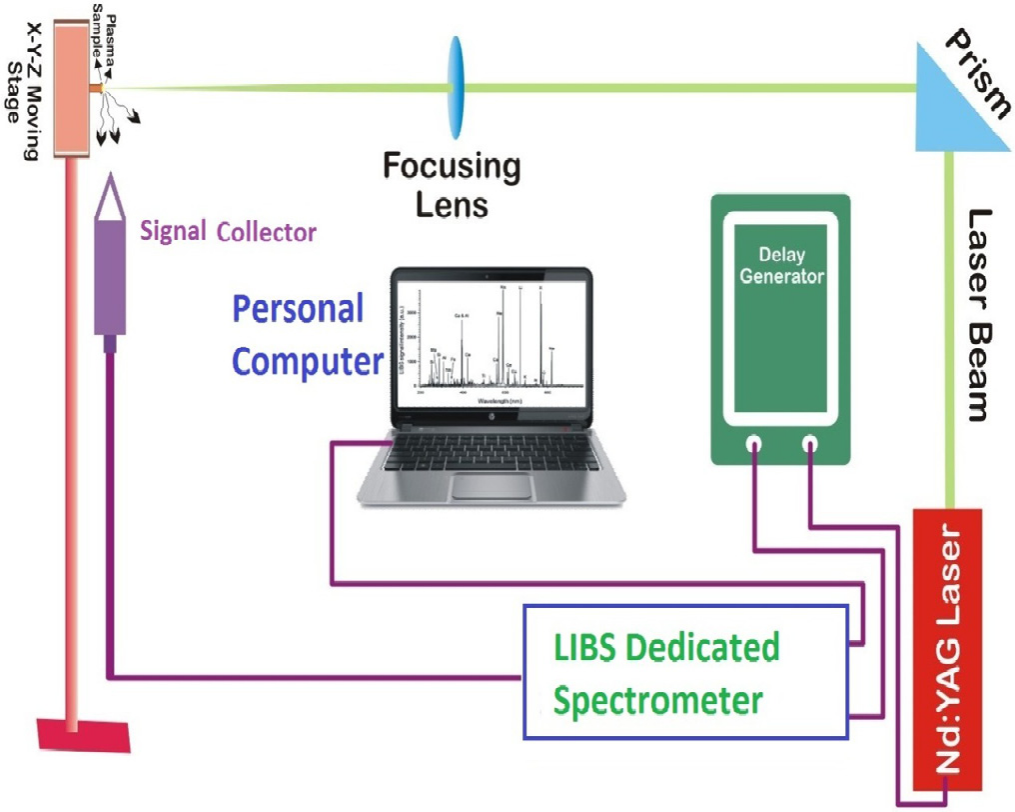
LIBS setup employed for elemental analysis of irrigated soil samples.

The schematic of the LIBS experimental setup applied in the present work is depicted n Fig. 1**.** It consisted of a pulsed Nd-YAG Laser (Ocean Optics, Inc.) equipped with C.C.D. detector, bundle of fiber optic, convex lens, moving stage along with online computer.

A focusing lens of 20 cm focal length was used to focus a pulse signal on the plane pelletized samples which is placed on translational stage. The spectral emissions from the LIBS generated plasma was registered by fiber optic (high-OH, core dia: 0.6-mm) with a collimating glass (0°–45° field of view) located in vertical direction with respect to plasma development.

A flash lamp Q-switch delay via the laser controller was used to fluctuate the signal energy ranging from (20-to-140) mJpulse^-1^ and determined by a calibrated joule-meter. The experiments were performed at ambient environment conditions. The samples were mounted on a rotary stage which was rotating continuously during the experiment at uniform speed so that each laser shot hit fresh surface and to evade the creation of any crater on the sample surface.

To collect the LIBS spectra, the detector and the Q-switched of the laser system were harmonized with a four-channel digital pulsed generator. The time span between the signal firing and the opening of the detector was adjusted to reduce the involvement of the continuum emission and the selection of time delay of ~ 3.5 μs was observed the best setting. All sorts of electronic disturbance (e.g., C.C.D. dark current) were acquired individually and got removed from the spectra for the investigated samples. Each spectrum was acquired utilizing a single shot mode and then taking the average for 20 such laser shots to minimize the statistical errors. Lastly, the resultant spectra were identified and marked the elements which are present in the test sample along with finger print wavelengths using commercial statistical software (Origin Pro-8.0).

### 2.1 Sample collection area in this study

In the present work, the soil collection selected area was the agricultural land located in the KP province of Pakistan. The selection of the area was mainly based on the cultivated land which utilizes different water sources for irrigation the crops. It is worth mentioning that various industrial units are also situated in the nearby area of the selected region. In this area, abundant acres of cultivated land are irrigated by means of industrial waste water. Similarly, some of the agricultural land has different means of irrigation sources like water from canals, tube wells, and some acres of land which rely only on the natural rain water.

### 2.2 Sampling of soils and analysis

A set of four soil samples from cultivated land of different irrigation water were selected. The chosen soil samples were acquired through hand spade depths of 10–20 cm, first sieved and then dried under ambient environmental conditions under sun for four days. For complete removal of the humidity, the samples were dried in an oven at moderate temperature at finite temperature. The dried samples cleaned and crushed to fine powder (~200 mesh, screen ~ 75 μm) for the LIBS analysis. The fine powder of soil samples were placed in air tight glass bottles and were coded as tabulated in Table 1.

**Table 1:** List of cultivated soils analyzed in this work.

**Sample Codes**	**Collection based on irrigation water**
Sample 1	Industrial water based cultivated soil
Sample 2	Tube well water based cultivated soil
Sample 3	Lake water based cultivated soil
Sample 4	Rain water based cultivated soil

To examine the cultivated soil for toxic and major nutritional elements using ICP-OES, we performed the typical working procedure (T.W.P.) for digestion and examination approved by Environmental-and-Protection Agency (E.P.A.). For this purpose, the cleaned powdered cultivated soil samples were first treated with 5% HNO_3_ (~99.99% pure) in a block heater made of graphite at ~ 94 °C, later on by the substitution of H_2_O_2_ and was allowed to complete exothermic reaction and then refluxed at ~ 95 °C with concentrated HCl. After, the mixture was passed through a filter paper and studied using ICP-OES spectrometer (OPTIMA 2100-DV; PerkinElmer, Twofold View).

For LIBS measurement, about 1.8 gm of the fine powder of each soil samples were taken and pressed to pellets in dimensions of 2-cm dia and 1-cm thickness through a hydraulic press by application of pressure of ~ 20 ton for a time period of 30 min. The pellets of samples were exposed to an energetic laser beam to generate the plasma plume. Luckily the pallets were so firm to withstand the power of incoming laser beam without the use of any binding material.

## 3 Results

### 3.1 The optimization of LIBS parametric quantities

The subsequent sections discuss the optimization of the LIBS parameters to acquire the best signal-versus-noise (S-N) ratio which is incredibly critical in LIBS quantitative determinations.

#### 3.1.1 Selection of laser energy

Numerous emission spectra of the collected soil samples were acquired for pulse laser energy in the 20–140 mJ/pulse. The integrated signal intensity for Mg-I (518.35)-nm line versus the selected laser energies was studied. It was noticed that the integrated signal intensity was linear dependent on the focused laser energy which is evident through the highest R^2^ fit value. Since, at higher incident laser energy, we recorded higher LIBS signal intensity which leads to the generation of an optical thick plasma with high back-ground continuum, which jeopardize the content determination accuracy. Consequently, we kept the pulsed energy at 60 mJpulse^-1^ for the existing work and it was found that 60 mJpulse^-1^ was sufficient to get the requisite LIBS signal intensity.

#### 3.1.2 Selection of focusing point of convex lens

The emission spectra were acquired through focusing the laser beam with a convex lens of focal distance of 10-cm by variable focal spaces (07, 08, 09, and 10) cm. It was observed that when the targets were placed at various distances from convex lens, the observed peak intensities of the discharging elements were powerfully variable, suggesting thereby un-stable plasma. The mainly exceptional choice was established to be when the sample under test was placed at distance of 9.0 cm from the lens; the emitting peaks acquired were of stable heights.

Similarly, the time span between laser excitation and acquirement of the plasma emission is very important to get spectra that reflect the spectral characteristics of the elements present in the sample plasma. At the primary stage, the plasma is so warm with high density of ionized species that the emitted light at this phase will lead to a featureless continuum emission. Moreover the collecting the plasma emissions at later states will provide very low fluorescence due to total decay of the neutral atomic emissions. Consequently, there exists an optimum time span between the excitation by laser and the attaining of neutral lines emissions, where the resultant spectrum depicts the chemical composition of the target sample with optimum LIBS spectral intensity. In the present work, the optimum time delay was predicted through repeating the scan by varying the time delay between 0 μs to 5.0 μs with an increment of 0.5 μs and the gate delay of 3.5 μs was found astonishingly appropriate for the recording of the best LIBS peak intensity with minimum background. The optimized parameters were used to obtain the LIBS spectra of the target samples.

### 3.2 Spectroscopic study of soil samples

After having known the optimized experimental parameters for the present analysis, LIBS emission spectra were recorded in the 220–800 nm wavelength region to carry out qualification and quantification of essentials there in the cultivated soils targets. The optical fiber was positioned at a length of ~ 0.1 cm from the plasma plume.

**Fig. 2:**
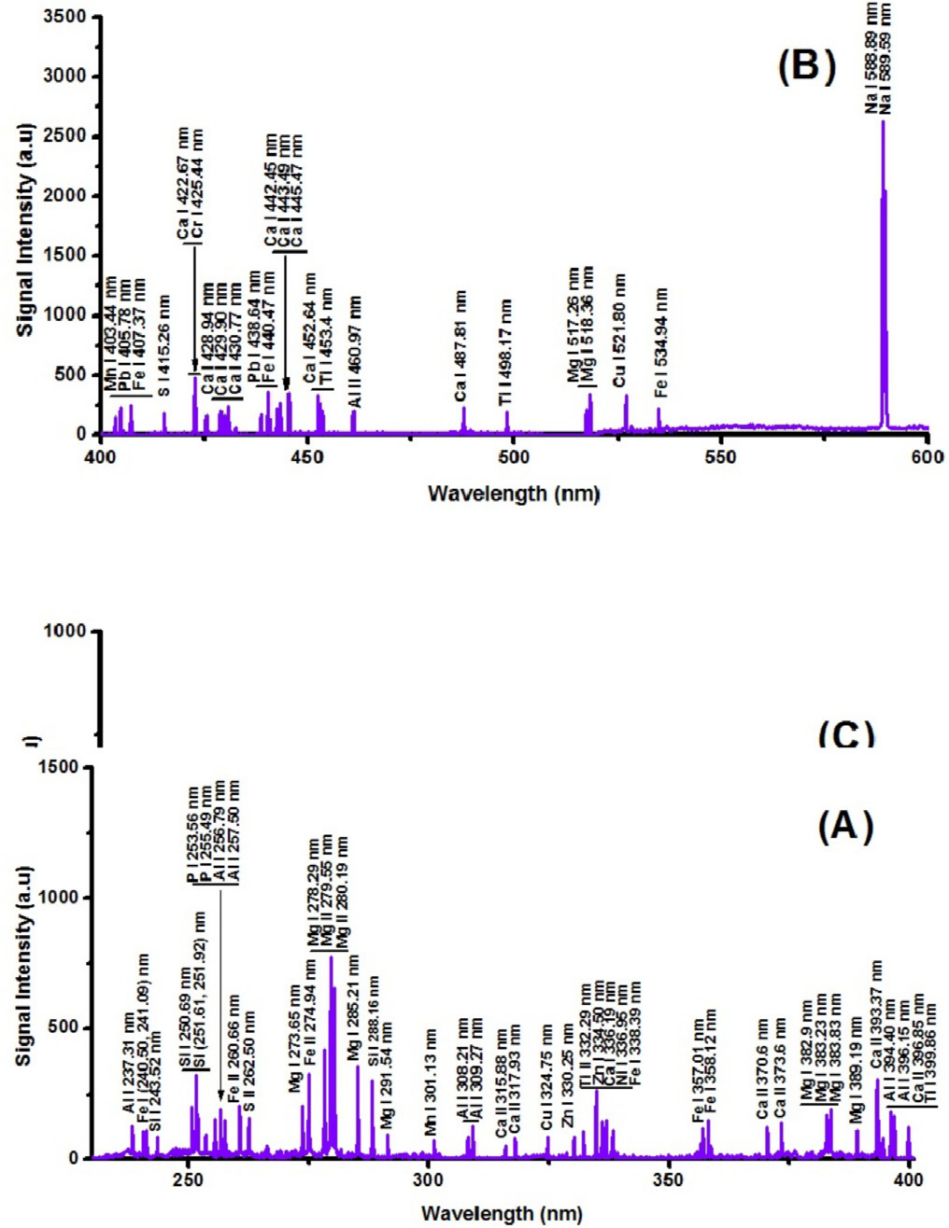
**(A-C):** Representative LIBS spectrum of sample-1.

The LIBS plasma spectrum of cultivated soil sample (sample-1) is presented in Fig. 2 **(A-C)** in 230–800 nm wavelength range. All the elements were recognized by the NIST atomic database. The identified wavelengths linked to different species there in cultivated soil targets are evidently apparent from less back-ground peak intensity. In general, the strong emission peaks belong to chief constituent elements, whilst the weak peaks belong to trace elements. The recorded spectra of all soil samples were very rich, containing singly ionized ions (II) and atomic species. Most of the emission lines are in the ultraviolet and visible wavelength range beside the Hydrogen (H_α_) line at 656.2 nm. Here spectra of all selected samples exhibit the existence of aluminum (Al), iron (Fe), silicon (Si), magnesium (Mg), manganese (Mn), calcium (Ca), zinc (Zn), nickel (Ni), lead (Pb), copper (Cu), chromium (Cr), lithium (Li), silver (S), Titanium (Ti), potassium (K). We also observed atmospheric lines of nitrogen (N) and oxygen (O) in the NIR region of emission spectra.

### 3.3 Calibration free (CF)-LIBS approach

The algorithm of calibration-free LIBS suggested by Ciucci et al. (Ciucci et al., 1999), based on the supposition of stoichiometric ablation, state of local thermodynamic equilibrium, and optical thin plasma was developed. The segments below demonstrate the validity of such assumptions for the laser-generated plasma in our LIBS analysis of cultivated soil.

#### 3.3.1 Stoichiometric ablation

As a powerful nanosecond laser signal is focused at the target, due to the heating and expansion of the exposed material, it attains the vaporization phase as it results into the surface burst earlier than the surface layer can vaporize; consequently the promptly heated materials have the similar formation as the compacted form intending to stoichiometric ablation. In this work, laser ablation of the soil targets was obtained by first harmonic of ND-YAG laser @ 1064 nm, pulsed interval of about 5-ns and a repetition rate of 10 Hz. With a spot size of about 20x10^3^ nm and laser energy of 60 mJ, the corresponding power density was found to be 3.0 × 10^12^ W/cm^2^ thereby satisfying the state of stoichiometric ablation (Rehan et al., 2018).

#### 3.3.2 Self-absorption

One of the important aspects in implementing the spectral emission intensities to determine the elemental concentration in LIBS is to make sure the condition of optically thin plasma i.e. dimensions and formation of the plasma should be such that the radiations emitted by the excited plasma species are not to be reabsorbed by any other atomic species containing comparatively lower level of energy. The effect of self-absorption can be simply verified using the ration of intensity of two interference-free spectral peaks from an element with the identical higher energy state such that this intensity ratio will furnish same value as the corresponding transition probabilities ratio value in case of the absence of self-absorption.

Particularly, to determine the presence of self-absorption, we made a comparison between the intensity ratio of the non-resonant peaks of Ca and Mg for all samples with the corresponding transitional probability ratio (see Table 2). Interestingly, the said ratios were in great agreement. The regularity between the ratios of intensity and the ratios between consequent transitional probabilities (with R.S.D. ± 10%) obviously exhibits the presence of thin plasma dimensionality.

**Table 2:** Table showing comparison in the ratios of non-resonant peaks and their analogous transition probability ratios using sample-1.

	**Sample-1**	
	Ratio between LIBS Signal Intensities	Ratio between transition probabilities
Ca (428.94)/Ca (429.90)	1.23	1.29
Mg (382.9)/Mg (383.2)	1.33	1.39

### 3.4 Measurement of plasma parameters

To diagnose the plasma contents and to get the valuable information, correct estimation of the basic plasma parameters like electron number density N*_e_* and plasma temperature (T) are the foremost prerequisites. Normally to estimate the value of T for the laser created plasma, the most common method considered is the Boltzmann plot method, which basically refers to a linear appearance of Boltzmann distribution, whereas the N*_e_* is estimated from the stark broadened profile of well-isolated and well-resolved emission line of a particular specie. To select the spectral emissions for calculating plasma parameters certain factors are to be taken into account. The first condition is that the plasma must be in the condition of Local Thermodynamic Equilibrium (L.T.E.) and the selected peaks should be thin optically and without self-absorption. Following the Boltzmann distribution of plasma species, the integrated line intensity of a emission line is given as (Aldakheel et al., 2020, Tognoni et al., 2007, Rehan et al., 2019, Gondal et al., 2012, Hussain and Gondal, 2008)

(1) $$I^{\mathrm{ki}}=FNA_{\mathrm{ki}}\frac{g_{k}}{U_{s}\left(T\right)}e^{-ek/kT}$$

where F is the experimental factor, N signifies the number density of emitting specie (atomic or ionized), g_k_ denotes degeneracy of the upper state k, A_ki_ represents the transition probability, E_k_ is energy of upper state, k is Boltzmann constant, T provides the electron temperature and $U_{s}\left(T\right)$ represents the partition function. Equation (1) is solved as,

(2) $$ln\left(\frac{I^{\mathrm{ki}}\lambda^{\mathrm{ki}}}{{\mathrm{hcA}}_{k}g_{k}}\right)=ln\left(\frac{\mathrm{FN}}{U_{s}\left(T\right)}\right)-\frac{E_{k}}{\mathrm{kT}}$$

This equation is a normal representation of a straight line, which can readily provide a plot of $ln\left(\frac{I^{ki}\lambda^{ki}}{{hcA}_{k}g_{k}}\right)$ against the energy of upper state levels (E_k_) of the perceived spectral emissions with a slope (−1∕kT) and an intercept, $q_{s}=ln\frac{{F.C}_{\sigma }}{U_{\sigma }\left(T\right)}$. The electron temperature can be obtained using the slope of the Boltzmann plot while the elemental abundance can be acquired from the line intercept (Tognoni et al., 2007) Fig. 3. As the amount of peaks of calcium exists in each spectra accordingly well-isolated and optically narrow lines of Ca (neutral as well ionized) were utilized to approximate the value of T. Using the atomic peaks of Ca, the spectral peaks utilized were 336.1 nm, 443.5 nm, 452.6 nm, and 643.9 nm. Similarly, for singly ionized we used Ca II at 393.3 nm, 396.8 nm, 370.6 nm, and 373.6 nm. The spectroscopic data of the determined spectral peaks were acquired from the online available database of NIST.

**Fig. 3:**
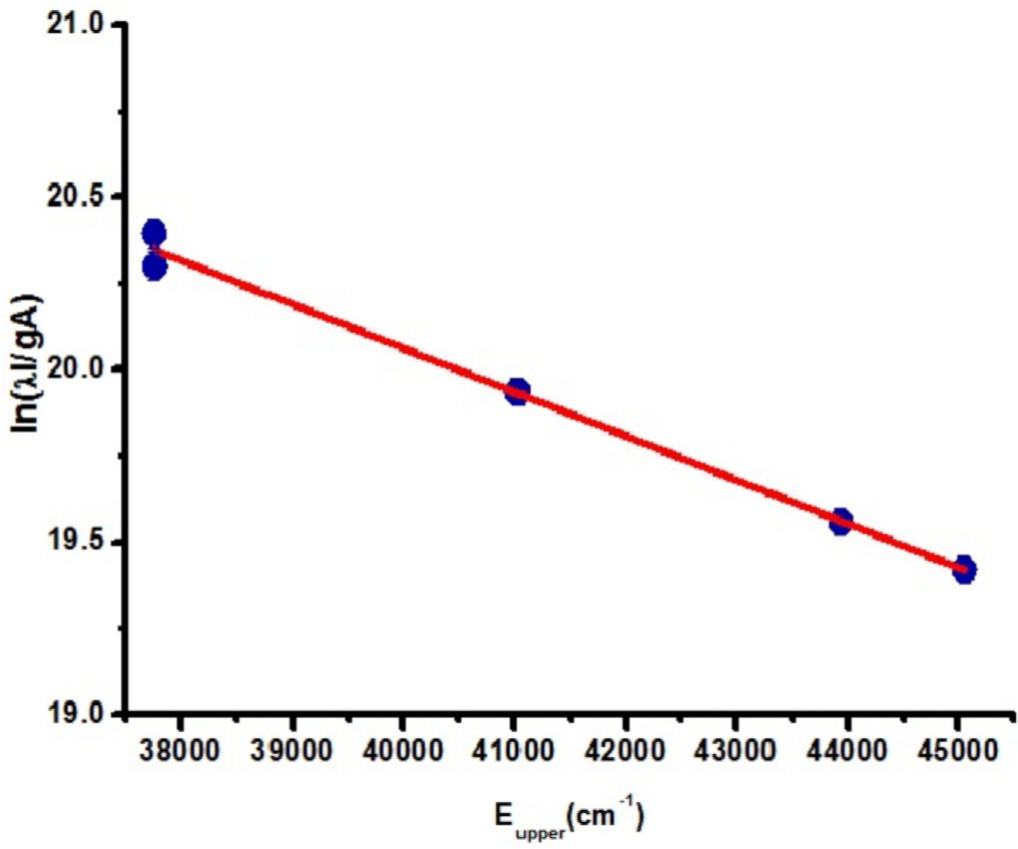
Boltzmann plot utilizing Ca I emission lines of sample 1.

Slope of the line, determined from the characteristics Boltzmann plot, provides the T as ~ 7800 K. Additionally we obtain the T using Ca-II lines, which in our case reveals the value electron temperature of about 7600 K. Similar to all the real life experimental investigations errors are unavoidable as here in the assessment of the electron temperature using the scheme of Boltzmann plot. Therefore, the value of T was calculated under the un-certainty of ± 10-% error, which in general accounts for accumulation of ambiguity in transition probabilities and in finding the integrated intensities of spectral lines. Average values of T for all cultivated soil samples were found to be in the range of (7800–9300) K.

**Fig.4:**
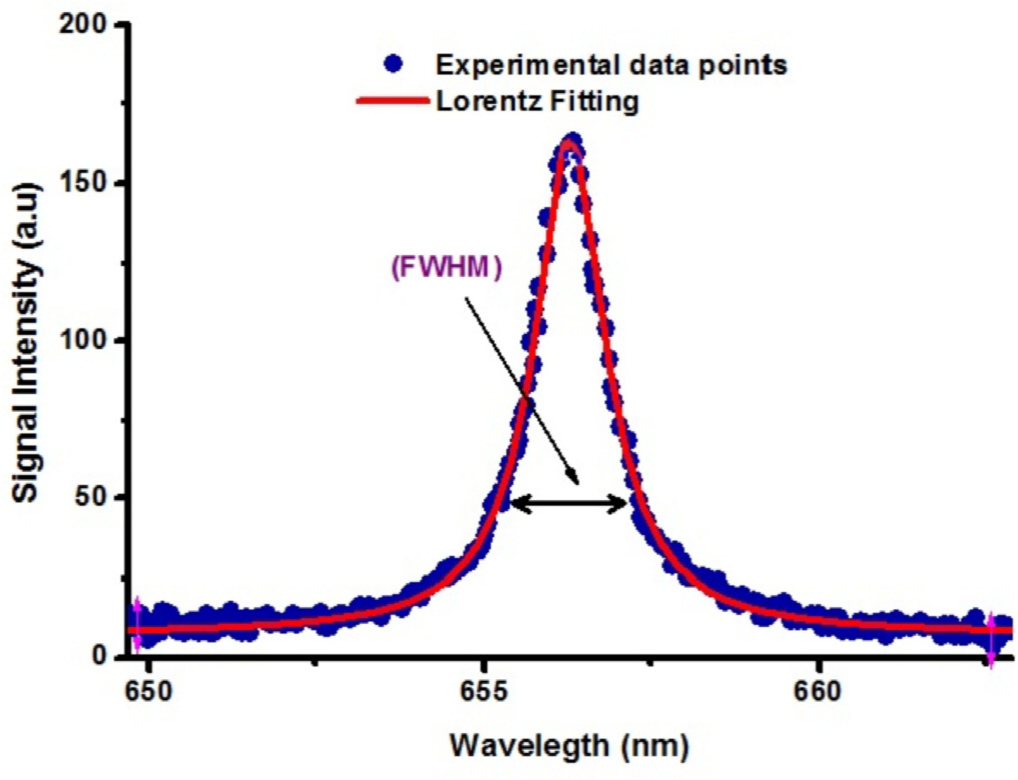
Lorentzian fit using stark broadened shape of atmospheric (H_α_) line at 656.27 nm.

Normally the N*e* is measured via the estimation of the widening of a well-isolated and well-resolved peak from the emission spectrum. The emission peaks may get broadened because of the effect known as self-absorption but we chose the self-absorption’s free spectral lines. The Stark widened line profile of H-alpha peak (656.27-nm) was used to quantify the full width at half Maximum (F.W.H.M.) through the Lorentzian fit of the experimental data as shown in Fig. 4**.** The relation between F.W.H.M. of H_α_ line and n*_e_* is given as

(3) $$n_{e}={\left(\frac{\lambda_{1/2}}{1.098}\right)}^{1.473}\times 10^{17}\left({\mathrm{cm}}^{-3}\right)$$

Using the value of F.W.H.M. for H_α_ line, the n*_e_* was measured ~ 1.11 × 10^17^ cm^−3^.

With the same approach, the values of N*_e_* for different selected cultivated soil samples were found in the range (1.11–1.60) × 10^17^ cm^−3^.

The stark broadening line profile of Ca – I at 422.67 nm was obtained utilizing Lorentzian fitting to the experimentally obtained data points and used to estimate its number density. The number density was determined as

(4) $$n_{e}\left({\mathrm{cm}}^{-3}\right)=\left(\frac{\Delta \lambda_{1/2}}{2w}\right)\times \exp^{16}$$

where ${\Delta \lambda }_{1/2}$ (nm) provides the F.W.H.M., and ω provides electron impact parameter in (nm). The measured value of $n_{e}$ was found as 1.2 × 10^17^ cm^−3^. Remarkably, the number densities estimated using the both lines (H_α_ and Ca) were in outstanding conformity. Hence in our case, the essential condition for L.T.E. plasma existence is well satisfied for the above mentioned values of T*_e_* and *n_e_* under the McWhirter criteria as described below.

### 3.5 Local thermodynamic equilibrium

As mentioned above, one of the indispensable requirements to be satisfied with Boltzmann plot method is that the plasma must be in the L.T.E. So as to confirm the condition of L.T.E., we have to achieve with the McWhirter’s criterion (Rehan et al., 2019) as,

(5) $$n_{e}\left(cm^{-3}\right)\;⩾1.4\times 10^{14}{\left(T\right)}^{1/2}\left(\mathrm{eV}\right){\left(\Delta E\right)}^{3}\left(\mathrm{eV}\right)$$

where T (eV) refers to the electron temperature, ΔE signifies the difference in energy states being concerned (eV). In the present case, the electron number density was measured by the H_α_ line. Incorporating the value of energy ΔE (eV) for H_α_ (1.889 eV) and measured electron temperature (eV), provides electron density of about ~ 7.73 × 10^14^ cm^−3^. The estimated value of *n_e_* was greatly higher than the limit thus entailing the confirmation of L.T.E. supposition.

## 4 Discussions

### 4.1 Quantification of soil samples

#### 4.1.1 Free calibration (CF)-LIBS method

In the present study to carry out the compositional study of each cultivated soil samples incorporates the one-line calibration free CF-LIBS method (Ciucci et al., 1999). The extant of precision of the method is reliant upon the accurate measurement of plasma parameters (electron temperature (T), and electron number density (*n_e_*)). In this methodology the Boltzmann relation was used to find out the amount of atomic species in the plasma can be written as,

(7) $${\mathrm{FC}}_{s}=I^{\mathrm{ki}}\frac{U_{s}\left(T\right)}{A_{\mathrm{ki}}g_{k}}e^{\mathrm{ek}/kT}$$

where $I^{\mathrm{ki}}$ stances for the integrated intensity of an emission line, C_s_ denotes the quantity of neutral elements available in the plasma, F is experimental factor which is related to the mass ablated material, $A_{\mathrm{ki}}$ refers to the transition probability, g_k_ stands for statistical weight, E_k_ represents the energy of higher state, k represents the typical Boltzmann constant, T symbolizes the electron temperature, and U_s_(T) is the partition function. Following the determination of densities corresponding to neutral atoms, the densities of ionized atoms were determined by utilizing the Saha-Boltzmann equation (Ciucci et al., 1999). However, the total amount of species in the sample can be obtained by summing the contributions of the atomic as well as the ionized species as,

(8) $$C_{s}^{t}=C_{s}^{z}+C_{s}^{z+1}$$

Employing the above procedure, we calculated the abundances of all the elements in each of cultivated soils.

#### 4.1.2 Standard calibration curve (CC)-LIBS method

**Fig. 5:**
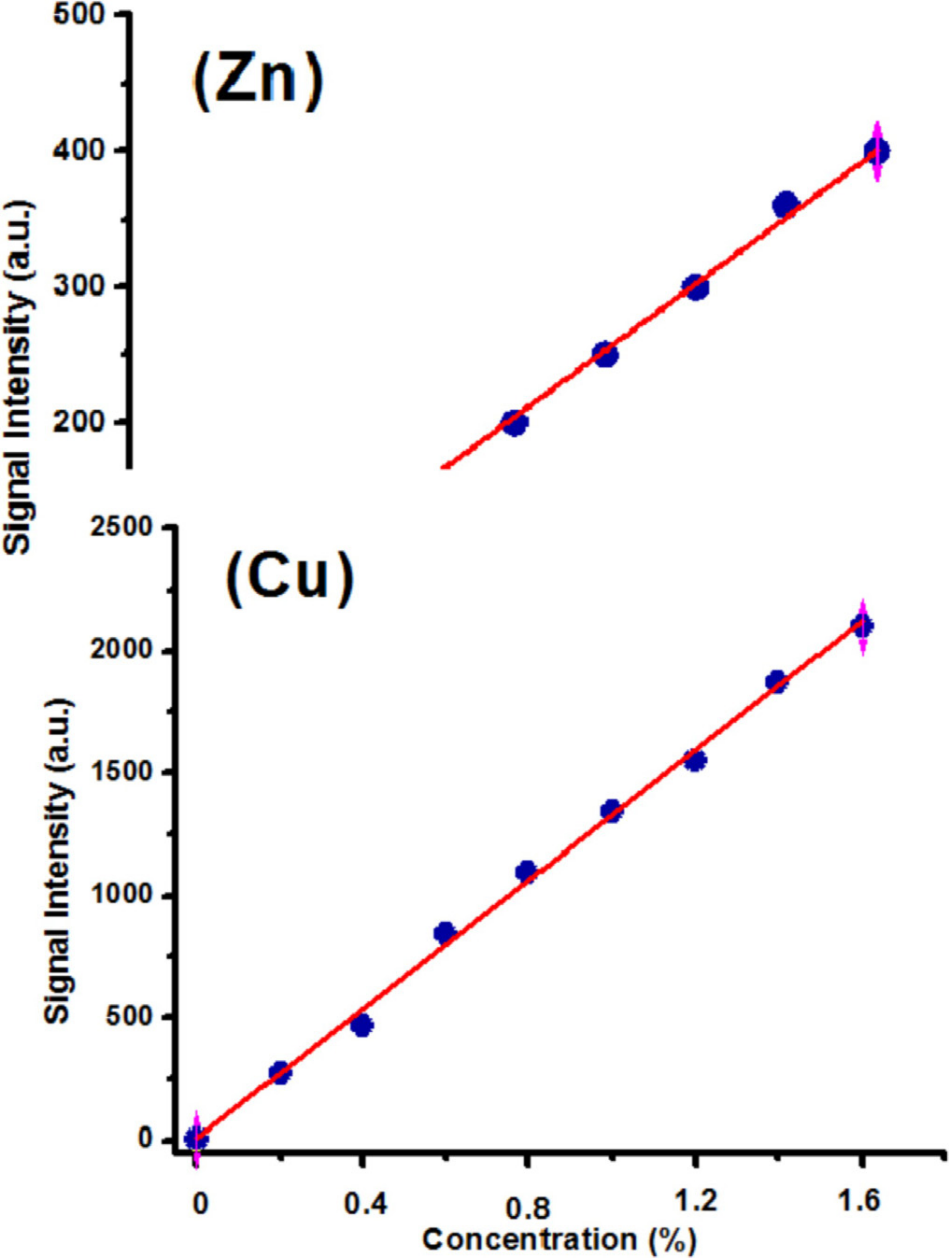
Typical calibration curves for Cu, and Zn present in sample 1.

A very frequent application of LIBS consists in determining the abundance of a species in a given sample by using the calibration curves. In our study, we have also employed the standard calibration curve (CC)-LIBS approach to perform the quantitative study of the selected cultivated soil samples for the determination of essential and toxin contents. This method relies upon the plot of a graph between the known amounts of detected elements against their LIBS generated signal intensities (calibration curve). All of the observed elements were obtained in ~ 99.99-% pure fine powder forms. To perform quantitative investigation of the species of importance exist in the cultivated soil samples, the relevant standard calibration curves were drawn by means of species of known amounts in the target matrix (soil). The abundance of a particular constituent in the typical calibration sample and their relevant LIBS signal samples provides a relationship linking the spectral peak intensity and the unidentified amount of elements. To be sure to have excellent mixing and uniformity, powder form of all of the observed species were mixed with the respective cultivated soil matrix in a ball milling apparatus. Various typical samples i.e. 0.002-%, 0.2-%, 0.4-%, 0.8-%, 1-%, 1.2-%, 1.4-%, and 1.6-% of the observed species were arranged in cultivated soil matrix. The LIBS generated peak intensities from typical samples were acquired and plotted versus the relevant quantities (%). The typical calibration curves of Cu and Zn for sample 1 are presented in Fig. 5**.**

The calibration curves have highest linear regression coefficient (R^2^), thereby giving reliable quantitative analysis. The determined values of essentials and toxic metals in all of the studied samples using CF-LIBS, CC-LIBS, and ICP-OES are tabulated as in Table 3a, Table 3b.

**Table 3a:** The abundance of the essentials elements as measured in all of the selected cultivated soil samples using CC-LIBS, CF-LIBS and ICP-OES methods.

Elements observed	CF-LIBS (%)	CC-LIBS (%)	ICP-OES (%)
**Sample 1**			
Al	12.0	11.7	11.5
K	9.0	8.70	8.50
Ca	9.0	9.10	9.20
Fe	10.0	10.2	10.3
Mg	8.0	8.10	8.20
P	6.0	6.10	6.20
Na	6.50	6.30	6.20
S	9.0	9.40	9.40
Si	7.0	7.10	7.20
**Sample 2**			
Al	15.600	15.4	15.3
K	10.595	10.8	10.7
Ca	10.500	10.2	10.2
Fe	11.500	11.8	11.7
Mg	11.500	11.7	11.996
P	11.500	12	11.9
Na	6.4000	6.8	7.0
S	4.0000	3.7	3.6
Si	7.4000	7.0956	7.2
**Sample 3**			
Al	13.57	14.1	14
K	10.65	10.7	11
Ca	11.5	11.8	12
Fe	11.41	11.5	11.7
Mg	10.163	10.5	10.4939
P	9.5	9.8	9.7
Na	6.0	6.7	6.5
S	5.0	4.5	4.7
Si	7.50	7.2	7.0
**Sample 4**			
Al	14	13.4965	14.5
K	10.5	10.9	11
Ca	10.5	11	11.2
Fe	12.496	13	13
Mg	10.5	11	10.8
P	7.5	7.9	8.0
Na	6.5	6.3	6.0
S	4.0	3.8	3.8
Si	7.5	7.0	7.097

**Table 3b:** The abundance of toxins measured in the studied cultivated soil samples using CC-LIBS, CF-LIBS, and ICP-OES methods.

Elements observed	CF-LIBS (%)	CC-LIBS (%)	ICP-OES (%)
**Sample 1**			
As	1.5	1.6	1.7
Cr	2.0	2.1	2.2
Cu	2.5	2.6	2.7
Ni	2.5	2.2	2.3
Mn	3.0	3.0	3.1
Sn	2.0	1.9	1.8
Ti	2.0	2.0	1.8
Zn	3.0	3.2	3.2
Ba	5.0	4.7	4.6
**Sample 2**			
As	1.1	1.0	1.0
Cr	0.002	0.0017	0.0015
Cu	1.5	1.4	1.3
Ni	2.0	2.1	2.2
Mn	2.0	2.0	1.8
Sn	4.0	3.7	3.6
Ti	0.8	0.7	0.7
Zn	2.1	2.0	2.0
Ba	0.003	0.0027	0.0025
**Sample 3**			
As	1.6	1.4	1.4
Cr	1.0	0.9	0.8
Cu	2.0	1.9	1.8
Ni	2.0	1.8	1.8
Mn	2.5	2.3	2.2
Sn	1.0	0.8	0.8
Ti	1.6	1.4	1.4
Zn	3.0	2.6935	2.7
Ba	0.007	0.0065	0.0061
**Sample 4**			
As	1.7	1.6	1.5
Cr	1.5	1.7	1.4
Cu	2.3	2.2	2.0
Ni	2.3	2.2	2.0
Mn	2.7	2.4	2.3
Sn	1.5	1.3	1.4
Ti	1.0	0.9	0.8
Zn	3.5	3.3	3.2
Ba	0.004	0.0035	0.003

From chemical composition of the selected soil samples it is quite clear that in cultivated soil collected from the industrial area, toxic metals like Chromium, Arsenic, Copper, Nickel, Manganese, Titanium, Zinc, and Barium was observed in most abundant beyond the permissible limits. A glance on Table 3b shows that the cultivated land using tube well water contains toxins in least amount as compared to all the other samples. Similarly, in term of essential species, the sample 2 was very rich. In sample 1, sulphur was found to contain the largest amount ~ (9.0%) far above the maximum permissible limit of sulphur. Similarly, the amount of chromium (2.0%), and Barium (5.0%) were also observed far above the maximum permissible limits of these elements. It is interesting to mention that tube well water based cultivated soil was relatively rich of nutritional elements like Calcium, Magnesium and Potassium whereas least amount of toxic metals like Aluminum, Chromium, Arsenic, Nickel, Copper are found. From these results one may conclude that for agricultural purpose, tube well is the best source of irrigation water for the cultivated soil and for the crops grown in the soil. For the sample 3, and sample 4, the contents of essentials and toxic metals were found between sample 1, and sample 2. The toxins frequently lead to deprivation of soil health and pollution of food chain mostly via the vegetables grown on such cultivated soils, since, the method of metal intake and gathering by various plants is dependent on the contents of existing metals present in the soils. Lengthy waste-water irrigation may cause the storing of toxic metals in cultivated soils as well as in vegetables. Issues of food safety and possible health hazards create this as one of the prime dangerous environmental problems. Plants stock toxins in their edible as well as in non-edible parts. Though certain heavy elements like Cu, Zn, Ni, and Mn acts as micronutrients in lesser amounts, but they become toxic at greater contents. In the light of current outcomes and findings, it is highly recommended that industrial water must not be used for cultivation at all as out flux from the industry contained huge amount of toxic metals which are health hazardous for the underground water as well as for the crops grown in the area and hence for the human beings.

## 5 Conclusion

The present work explore the application of an optimized LIBS approach to carry out the elemental distributions in cultivated soil samples, more specifically pointing to estimate the impact of irrigating water on its chemical composition. Prior to its application for soil analysis, LIBS system was improved via analyzing the reliance of integrated LIBS peak intensity on experimental parameters. In order to determine accurate elemental contents of the selected samples, the calculations were performed under the prerequisite conditions of Local Thermodynamic Equilibrium and optical thin plasma which were verified using the McWhirter criteria. Furthermore, the effect of self-absorption was investigated via the electron number density related to Hα line. It is worth mentioning that the electron density (Ne) estimated using the Hα line of hydrogen was found to be in accordance with the value determined using neutral spectral emission lines of Ca, thus verifying the absence of self-absorption and hence our quantitative analysis appears more reliable and accurate. It is remarkable that the nutritional contents of cultivated soils were found strongly dependent upon the type of irrigation water or the water sources. In addition to the abovementioned facts, to cross check and validate the concentration of detected elements using our LIBS system, a standard technique known as ICP-OES was also applied. A comparative study revealed that both of the results obtained using LIBS and ICP-OES are in excellent agreement. This study is highly beneficial for increasing the crop yield and to avoid the presence of toxic elements in agricultural crops as well food products using various sources of water especially to avoid any industrial waste water for irrigation purposes in developing countries.

## Declaration of Competing Interest

The authors declare the following financial interests/personal relationships which may be considered as potential competing interests: There is no financial interests or personal relationships may be considered as potential competing interests.
